# Commentary: Photoplethysmography for Quantitative Assessment of Sympathetic Nerve Activity (SNA) During Cold Stress

**DOI:** 10.3389/fphys.2021.602745

**Published:** 2021-06-23

**Authors:** Stefan Ackermann, Sylvain Laborde, Uirassu Borges, Emma Mosley

**Affiliations:** ^1^Institute of Psychology, Department of Performance Psychology, German Sport University Cologne, Cologne, Germany; ^2^Normandie Université Caen, UFR STAPS, EA 4260, Normandy, France; ^3^Institute of Psychology, Department of Health and Social Psychology, German Sport University Cologne, Cologne, Germany; ^4^Department of Sport Science and Performance, Southampton Solent University, Southampton, United Kingdom

**Keywords:** photoplethysmography, heart rate variability, sympathetic nerve activity, cold stress, psychophysiology

In relation to the paper “Photoplethysmography for Quantitative Assessment of Sympathetic Nerve Activity (SNA) During Cold Stress,” we have recommendations that may be of importance when considering future research aiming to assess SNA with photoplethysmography (PPG). The aim of this study was to investigate the differences in SNA between the periphery and the core during the cold pressor test (right-hand immersion in ice water) and cold exposure (whole body exposed to cold air) using PPG. Based on significant changes in the Low Frequency (LF) to High Frequency (HF) (LF/HF) ratio, the authors concluded that PPG is an effective method to measure SNA. From this conclusion we have highlighted three main issues that need to be addressed. Firstly, the LF/HF ratio is used as evidence for SNA change, based on the assumption that LF reflects SNA, a concept that has long been rejected in psychophysiology. Secondly, given the organization of the sympathetic nervous system, it is not possible to talk about SNA as a single unique concept. Finally, frequency components are investigated without considering breathing frequency, which is very likely to change during the cold pressor and cold exposure test. These critical points are now discussed in detail below.

Regarding the first point, Budidha and Kyriacou (2018, p. 2) base their goal to assess SNA using HRV on four articles. “The methods commonly used to study SNA include [...] HRV analysis of ECG (Appelhans and Luecken, 2006; Thayer et al., 2012; Deng et al., 2018; Hodges et al., 2019) [...].” However, none of these papers support the author's interpretation of the LF/HF “to quantify the changes in the sympathetic activity” (Budidha and Kyriacou, 2018, p. 5), but refer to it as reflecting the HRV assessment of the “sympathovagal balance.” Previous research indicates that the LF/HF ratio does not bear a significant correlation with SNA (Saul et al., 1990; Kingwell et al., 1994; Van de Borne et al., 1997b). The LF band (0.04–0.15 Hz) is produced by both the sympathetic and the parasympathetic nervous system (Akselrod et al., 1981; Berntson et al., 1997). With primarily parasympathetic nervous activity *via* baroreceptors (Reyes del Paso et al., 2013), or baroreflex activity alone (Moak et al., 2007) being contributing factors, LF is suggested to reflect baroreceptor activity in resting individuals (McCraty and Shaffer, 2015), not SNA (Introna et al., 1995; Van de Borne et al., 1997a; Heathers, 2012, 2014; Billman, 2013). The latter has been clinically demonstrated using blockade studies (Goldstein et al., 2011; Rahman et al., 2011; Reyes del Paso et al., 2013; Heathers, 2014; Martelli et al., 2014). Moreover, while sympathetic re-innervation in transplanted hearts is associated with non-respiratory LF (Bernardi et al., 1998), LF might further be influenced by slow respiration rates through vagal activity (Ahmed et al., 1982; Brown et al., 1993; Tiller et al., 1996; Lehrer et al., 2003; Kromenacker et al., 2018). Finally, studies indicate that the contribution of SNA to LF varies depending on different testing conditions (Eckberg, 1983; Kember et al., 2001; Shaffer et al., 2014).

Regarding the second point, the paper suggests that there is a unique SNA that varies from periphery to the core vasculature in its degree of control over cutaneous blood vessels during cold exposure. However, when examining the organization of the sympathetic nervous system in detail, the “various categories of sympathetic fibers can be controlled independently. There is not a uniform ‘sympathetic tone' for all parts of the sympathetic system. High activity in some parts must coexist with low activity in others if the sympathetic system is to fulfill its tasks” (Brodal, 2010, p. 428). Hence, instead of a larger influence of SNA in the index finger compared to the ear canal, the respective results found in this study reflect two of the many different kinds of SNA. At a structural level, the sympathetic nuclei in the vertebrae T1–T2–4 are responsible for the circulatory control in the head and neck region, whereas the vertebrae T3–T6 manage the circulatory control in the upper extremity (Brodal, 2010). At a functional level, “higher levels of the CNS (such as the hypothalamus) can selectively control subdivisions of the sympathetic system” (Brodal, 2010, p. 428).

Finally, regarding the last point, evidence suggests that cold water immersion causes hyperventilation (Tipton, 1989; Eglin and Tipton, 2005; Mantoni et al., 2008). As mentioned by Hodges et al. (2019), the frequency domains of HRV are especially vulnerable to bias in cold pressor tests because of their dependency on the participants' breathing pattern. HF, calculated in the range 0.15–0.4Hz, is expected to reflect cardiac vagal tone when breathing frequency is comprised between 9 and 24 cycles per minute (Malik et al., 1996; Berntson et al., 1997; Laborde et al., 2017). These respiratory cycles may be altered during a cold pressor task and subsequently effect the measure taken. Additionally, the interpretation of LF, expected to reflect a mix of sympathetic, parasympathetic, and baroreflex activity (Malik et al., 1996; Berntson et al., 1997; Laborde et al., 2017) may also be impacted by the influence of the cold pressor test on respiratory frequency. Consequently, it appears essential to assess breathing frequency when aiming to interpret HRV-frequency values during the cold pressor and cold exposure tests.

In conclusion, we have three main recommendations for researchers in this area: firstly, they should be aware of the existence of diverse types of SNA, and choose the appropriate measurement site/method depending on their research question. Secondly, blockade studies (Hagerman, 1996; González et al., 2000; Millar et al., 2010; Castiglioni et al., 2011) are required to investigate whether PPG can actually index SNA. Finally, when researching the effects of cold water immersion, the respiratory frequency must be considered, to ensure thorough interpretation of frequency-domain indices.

## Author Contributions

SA wrote the first draft. SL, UB, and EM provided critical comments to improve the manuscript. All authors contributed to the article and approved the submitted version.

## Conflict of Interest

The authors declare that the research was conducted in the absence of any commercial or financial relationships that could be construed as a potential conflict of interest.
